# Broken sleep predicts hardened blood vessels

**DOI:** 10.1371/journal.pbio.3000726

**Published:** 2020-06-04

**Authors:** Raphael Vallat, Vyoma D. Shah, Susan Redline, Peter Attia, Matthew P. Walker

**Affiliations:** 1 Center for Human Sleep Science, Department of Psychology, University of California, Berkeley, California, United States of America; 2 Department of Medicine, Brigham and Women’s Hospital and Beth Israel Deaconess Medical Center, Harvard Medical School, Boston, Massachusetts, United States of America; 3 Attia Medical, PC, San Diego and New York City, United States of America; Washington University in St. Louis, UNITED STATES

## Abstract

Why does poor-quality sleep lead to atherosclerosis? In a diverse sample of over 1,600 individuals, we describe a pathway wherein sleep fragmentation raises inflammatory-related white blood cell counts (neutrophils and monocytes), thereby increasing atherosclerosis severity, even when other common risk factors have been accounted for. Improving sleep quality may thus represent one preventive strategy for lowering inflammatory status and thus atherosclerosis risk, reinforcing public health policies focused on sleep health.

## Abstract 2

Why does poor quality sleep correlate with cardiovascular disease? A large population-based study suggests that fragmented sleep contributes to atherosclerosis in humans by increasing inflammatory-related activity of white blood cells.

## Introduction

Sleep disruption is associated with atherosclerosis. Why is this? One potential pathway through which fragmented sleep causally triggers cardiovascular disease is via the up-regulation of inflammatory-associated white blood cells, which incite atherosclerosis [1,2]. However, the proposition that sleep fragmentation in humans is associated with atherosclerosis through the mediating influence of increased neutrophil and monocyte counts remains unexplored [3–5]. Moreover, that such a pathway is evident even when accounting for common contributing factors leading to atherosclerosis—such as age, sex, ethnicity, body mass index (BMI), smoking status, blood pressure, use of antihypertensive medication, sleep apnea, and insomnia—is similarly unknown.

Here, we address these unresolved questions. Specifically, we test the hypothesis that the impact of fragmented sleep on atherosclerotic pathology is governed, in part, through the novel mediating influence of increased neutrophil and monocyte levels and, furthermore, that this sleep-related disease pathway is robust when multiple alternate cofactors (disease mechanisms) are being controlled for.

To do so, we examined the association between sleep fragmentation (measured using 2 independent sources of objective data: polysomnography [PSG] and multiple nights of wrist-based actigraphy), white blood cell count, and in vivo measures of subclinical atherosclerosis in a diverse sample of the population (*n* = 3,305). The characteristics of the cohort, stratified by atherosclerosis severity category, are shown in Table 1, and the sleep parameters of the cohort are presented in S1 Table. The unadjusted bivariate correlations described in the following paragraphs are shown in S1 Fig.

**Table 1:** Participant characteristics by atherosclerosis severity (CAC category).

	Very low (CAC = 0)	Low (CAC = 1–100)	High (CAC = 101–400)	Very high (CAC ≥ 401)	*p*-Value
**Count**	367	333	210	200	*-*
**Age**	64.4 ± 8.0	68.3 ± 9.2	71.6 ± 8.8	73.5 ± 8.0	**<0.001**
**BMI**	29.7 ± 5.7	29.2 ± 5.5	30.0 ± 4.9	29.4 ± 5.4	0.397
**Male sex**	29.7%	50.8%	51.9%	64.0%	**<0.001**
**Race, white**	31.9%	35.4%	45.2%	52.0%	**<0.001**
**Race, African American**	36.2%	29.4%	22.9%	18.5%	**<0.001**
**Race, Hispanic**	31.3%	34.2%	31.4%	28.5%	0.580
**Race, Chinese**	0.5%	0.9%	0.5%	1.0%	0.873
**Smoking, never**	43.6%	44.4%	35.2%	30.0%	**0.002**
**Smoking, former**	48.2%	48.3%	55.2%	62.5%	**0.004**
**Smoking, current**	8.2%	6.6%	9.5%	7.5%	0.656
**Any hypertension medication**	43.9%	56.8%	66.2%	69.5%	**<0.001**
**SBP**	122.0 ± 21	122.4 ± 19	126.1 ± 24	125.4 ± 20	**0.045**
**DBP**	68.4 ± 9.7	68.6 ± 9.4	68.3 ± 11.2	67.7 ± 10.1	0.769
**WBC count**	5.8 ± 1.7	5.8 ± 1.5	6.4 ± 2.0	6.6 ± 4.1	**<0.001**
**Neutrophil count**	3.4 ± 1.4	3.4 ± 1.2	3.8 ± 1.5	3.9 ± 1.6	**<0.001**
**Monocyte count**	0.4 ± 0.2	0.4 ± 0.2	0.5 ± 0.2	0.5 ± 0.2	**<0.001**
**Sleep apnea**	7.9%	6.9%	7.1%	5.0%	0.703
**Insomnia**	5.2%	3.9%	3.8%	3.5%	0.745

Data are shown as mean ± SD for continuous variables, and as percentages for categorical variables. *p*-Values were calculated using one-way ANOVA for continuous variables and chi-squared test of independence for categorical variables. Significant *p*-values are denoted in bold. Only the characteristics of the participants included in the complete-case mediation analysis (*n* = 1,110) are reported. The underlying data can be found in the BioLINCC repository at https://biolincc.nhlbi.nih.gov/studies/mesa/.

**Abbreviations:** BMI, body mass index (kg/m^2^); CAC, Coronary Artery Calcification; DBP, seated diastolic blood pressure (mmHg); SBP, seated systolic blood pressure (mmHg); WBC, white blood cell (10e3/uL)

## Results

### Actigraphy

**Fig 1:**
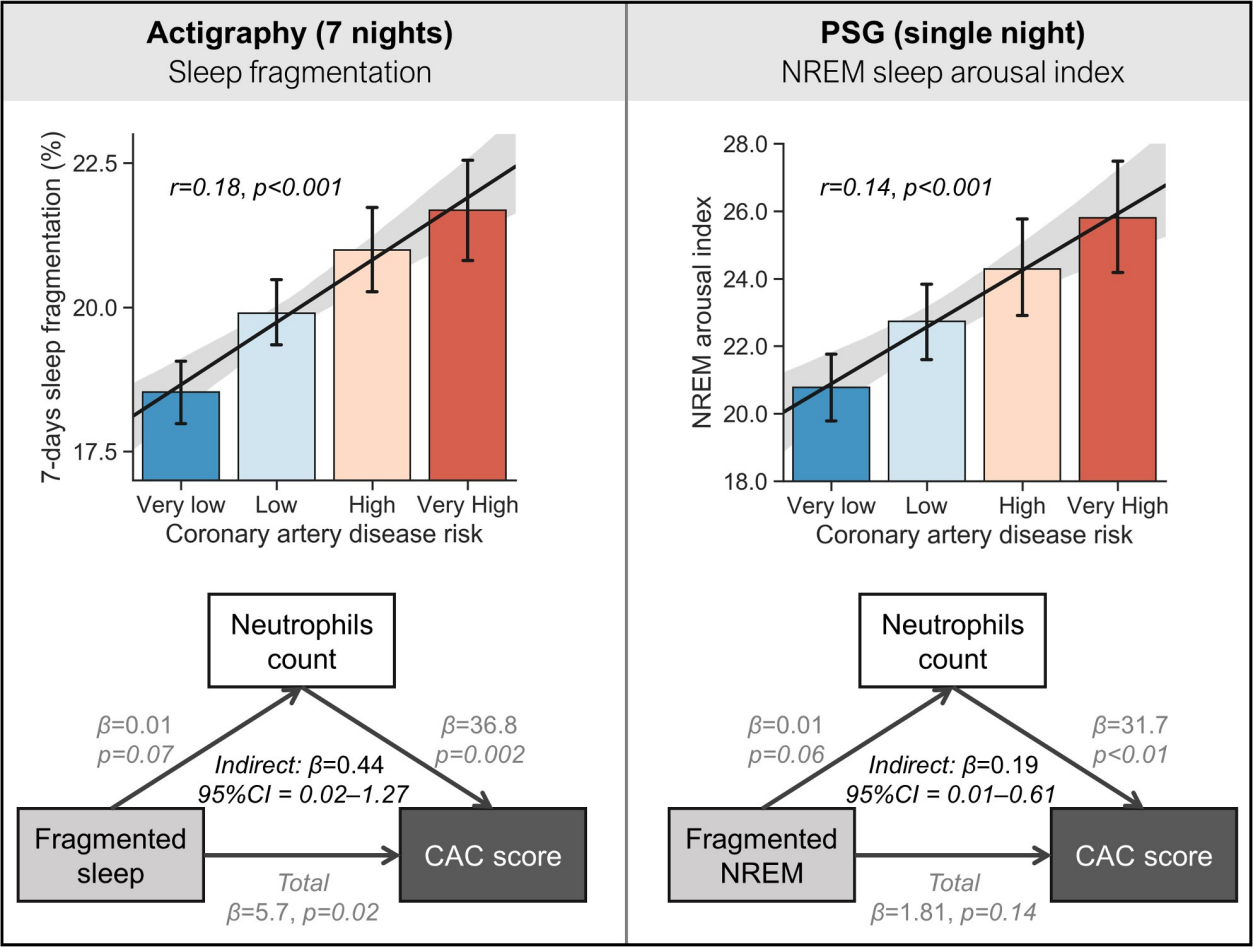
Results. (A) Actigraphy-measured sleep fragmentation is positively associated with coronary artery disease risk (Very low = 0 Agatston units, Low = 1–100, High = 101–400, Very high ≥ 401). Mediation analysis demonstrated a significant association between actigraphy-measured sleep fragmentation and increased absolute neutrophil count, which consequently predicted higher CAC scores. Thus, the link between fragmented sleep and atherosclerosis risk is, in part, governed by the impact of fragmented sleep on elevated neutrophils. (B) PSG measured sleep fragmentation (arousal index in NREM sleep) and the positive association with coronary artery disease risk. Here again, mediation analysis revealed a significant association between PSG-measured sleep fragmentation in NREM and increased absolute neutrophil count, which in turn predicted higher CAC scores. Cofactors controlled for in the mediation models included age, sex, ethnicity, BMI, smoking status, blood pressure, use of antihypertensive medication, as well as sleep apnea and insomnia diagnosis, described in the main text. The underlying data can be found in the BioLINCC repository at https://biolincc.nhlbi.nih.gov/studies/mesa/. BMI, body mass index; CAC, coronary artery calcification; NREM, non-rapid eye movement; PSG, polysomnography.

Focusing first on direct associations (prior to testing the mediation hypothesis and the inclusion of cofactors), actigraphy-measured sleep fragmentation positively and significantly predicted Coronary Artery Calcification (CAC) score (r = 0.18, *p* < 0.001; Fig 1A and S1 Fig). Second, this same objective measure of sleep fragmentation positively predicted higher neutrophil count (r = 0.08, *p* < 0.01) and was not significantly correlated with monocyte count (r = 0.04, *p* = 0.17). Third, both neutrophil and monocyte counts were positively associated with the CAC score (r = 0.12, *p* < 0.001 and r = 0.14, *p* < 0.001, respectively).

Having established each individual direct association, we next tested the central hypothesis that the relationship between sleep fragmentation and atherosclerosis pathology (CAC score) was not direct but, instead, statistically influenced through the indirect mediating impact of fragmented sleep on raised neutrophil count, which in turn predicted CAC score. Supporting this proposed pathway, the impact of sleep fragmentation on CAC scores was significantly mediated through the indirect pathway of raised levels of neutrophils (*n* = 1110, 𝛽 = 0.71, 95% CI 0.18–1.65).

Thus, sleep fragmentation was associated with atherosclerosis risk, yet this relationship was indirectly contributed to through the influence of fragmented sleep quality on increased neutrophil count. Consistent with the lack of a significant pairwise association between sleep fragmentation and monocytes, there was no indirect effect with monocyte count (𝛽 = 0.35, 95% CI −0.11 to 1.14), potentially suggesting a greater mediating role of neutrophil activity.

Numerous factors to date have been demonstrated to increase atherosclerotic risk, including age, sex, ethnicity, and BMI [6–8] as well as sleep-related features, including the presence of sleep apnea [9] and insomnia [10]. Importantly, the aforementioned mediation effect remained significant when controlling for the factors of age, sex, ethnicity, BMI, smoking status, blood pressure, and use of antihypertensive medication, as well as sleep apnea and insomnia diagnoses (𝛽 = 0.44, 95% CI 0.02–1.27; Fig 1A and S1 Methods). The mediation also remained significant when excluding participants with a CAC score of zero (*n* = 746, 𝛽 = 1.05, 95% CI 0.27–2.52) and in this more select cohort, again showed a significant mediation after controlling for all the aforementioned covariates (𝛽 = 0.85, 95% CI 0.11–2.36). Related, the mediation similarly remained significant when excluding 104 participants diagnosed with sleep apnea (𝛽 = 0.64, 95% CI 0.11–1.71, controlled for all aforementioned covariates).

Although in the same direction, the mediation effect was not statistically significant when adjusting for sleep apnea using the apnea-hypopnea index (AHI) estimated from the PSG night (𝛽 = 0.35, 95% CI −0.02 to 1.18). This indicates that sleep apnea cannot be excluded as a contributing factor in the mediation. However, post hoc analysis using AHI instead of sleep fragmentation as the exposure variable did demonstrate that there was no indirect effect of AHI on CAC via an increase in monocyte/neutrophil counts (neutrophil: 𝛽 = 0.12, 95% CI −0.02 to 0.44; monocytes: 𝛽 = −0.05, 95% CI −0.27 to 0.04). That is, sleep fragmentation, beyond AHI, appears to have a specific relationship with inflammatory-related increases in atherosclerosis.

### Polysomnography

Having quantified the association between atherosclerosis and home-based sleep, measured using wrist actigraphy, we further tested these same relationships using PSG-recorded sleep. Congruent with the actigraphy findings, the severity of PSG-measured fragmentation (arousals during non-rapid eye movement [NREM] sleep) directly and positively predicted CAC score severity (r = 0.14, *p* < 0.001). Once again, this association was indirect. Specifically, the impact of the PSG-measured arousal index in NREM fragmentation on CAC scores was mediated through raised levels of neutrophils (*n* = 1046, 𝛽 = 0.42, 95% CI 0.13–0.94) and raised monocytes (*n* = 1046, 𝛽 = 0.32, 95% CI 0.07–0.75). This effect was specific to NREM sleep, with no such significant associations with the arousal index measured during REM sleep (r = 0.003, *p* = 0.92).

The PSG-based mediation effect with neutrophils remained significant after controlling for age, sex, ethnicity, smoking status, and blood pressure (𝛽 = 0.19, 95% CI 0.01–0.61; Fig 1B). However, unlike the actigraphy-based measures, the effect did not remain significant after adjusting for BMI, sleep apnea, insomnia, and use of antihypertensive medication. One interpretation is that 1 week of wrist-based actigraphy sleep measurement, relative to a single night of PSG sleep recording, is more capable of detecting the sleep-dependent link between neutrophils and atherosclerosis when considering relevant cofactors.

The indirect mediation effect with monocytes did remain significant after controlling for age and ethnicity (𝛽 = 0.16, 95% CI 0.01–0.49), but not after adjusting for sex, indicating that the atherosclerotic impact of sleep fragmentation on monocytes (but not neutrophils) is partially regulated by sex.

It is noteworthy that the sleep-atherosclerosis mediation measured using PSG was significant for both neutrophils and monocytes, while actigraphy-measured sleep only showed a significant mediation effect with neutrophils (𝛽 = 0.36, 95% CI −0.11 to 1.14). This may suggest greater sensitivity of PSG measures in quantifying this atherosclerosis disease pathway with multiple inflammatory-related factors while still underscoring the aforementioned PSG results concerning comorbidities.

### Subjective sleep

Having examined the association between atherosclerosis and objective measures of sleep, we tested for an equivalent relationship using subjective reports of sleep fragmentation. Self-reported sleep fragmentation was not associated with neutrophil count (r = 0.009, *p* = 0.77), monocyte count (r = −0.056, *p* = 0.08), or CAC score (r = −0.042, *p* = 0.11) and provided no indirect mediation effect of the association between white blood cells and atherosclerosis (neutrophils: 𝛽 = 0.73, 95% CI −4.5 to 6.3, monocytes: 𝛽 = −4.3, 95% CI −10.7 to 0.11). In addition, there was no direct or indirect effect of habitual daytime sleepiness (measured by the Epworth sleepiness scale [11]) on CAC score. These findings indicate that, unlike objective assessments, self-reported sleep quality and daytime sleepiness may not offer statistically sensitive measures in the predictive mediation pathway between sleep, inflammation, and atherosclerosis.

### Exploratory analyses

Finally, we tested whether other objective sleep parameters, beyond sleep fragmentation, were similarly associated with atherosclerosis via an elevation in neutrophil and/or monocyte counts. Specifically, we looked at both actigraphy and PSG measures of sleep quantity and quality. Consistent with the aforementioned findings, PSG-defined wake after sleep onset (WASO) was indirectly associated with increased CAC through an increase in monocyte count (𝛽 = 0.05, 95% CI 0.01–0.13). Similarly, higher sleep efficiency (averaged across 7 days of actigraphy) negatively predicted a lower CAC score via a reduction in neutrophil count (𝛽 = −1.12, 95% CI −2.83 to −0.13). However, neither of these relationships remained significant after controlling for the earlier-mentioned cofactors. Thus, fragmented sleep, more than other sleep features, appears to be a particularly sensitive predictor of white blood cell–mediated atherosclerosis.

## Discussion

Together, these findings affirm a pathway in which the quality of human sleep, specifically the degree of fragmentation, raises inflammatory-related white blood cells, thereby conferring increased risk for atherosclerosis. This was true of sleep fragmentation assessed across a week or across a single night, which predicted increasingly higher CAC score through a mediating association with increased neutrophils.

Our findings confirm recent seminal work in mice demonstrating that experimentally induced sleep fragmentation, associated with increases in blood levels of monocytes and neutrophils, results in larger atherosclerotic lesions [1,2]. Furthermore, these rodent data added mechanistic insight, with sleep fragmentation reducing hypocretin levels in the hypothalamus, signaling bone marrow–related increases in the production of monocytes and neutrophils.

Advancing this research, we establish a sleep fragmentation—white blood cell—atherosclerosis association in a population-based sample of human adults and demonstrate that these effects remained robust when accounting for multiple other common atherosclerosis risk factors present in humans: age, sex, ethnicity, BMI, smoking status, blood pressure, and use of antihypertensive medication, as well as sleep apnea and insomnia diagnoses. Finally, we show that this indirect pathway can be quantified with objective sleep metrics, either using 1 week of wristwatch actigraphy or a single night of PSG recording.

Importantly, however, we demonstrate that this same disease sensitivity is not observed when using self-reported subjective sleep fragmentation or other metrics of sleep quantity and/or quality. This may be pertinent for clinicians and researchers in determining which sleep measures should be focused on in this disease context.

Though our statistical models remained significant after adjusting for age (in addition to other cofactors), this does not challenge the well-established and independent links between (i) aging and increases in monocytes and neutrophils [12,13], (ii) increases in atherosclerosis risk [14], and (iii) decreases in sleep quantity and quality [15]. Rather, our findings simply indicate that the mediation relationship between sleep fragmentation, white blood cells, and atherosclerosis persist when chronological age is considered.

Decreasing sleep duration and fragmented sleep are independently associated with an increased risk of atherosclerosis [16,17]. However, the pathways through which the impact of sleep impairment operates have remained largely unknown. Building on rodent models [1], our findings suggest that one candidate pathway through which sleep fragmentation can raise atherosclerotic risk in humans may be through raised levels of inflammatory-associated neutrophil and monocyte counts. This proposal is consistent with findings that insufficient sleep (acute and prolonged) triggers low-grade inflammation [18], decreases and increases in discrete immune factors, and enhanced upstream signaling mechanisms of inflammation, including those regulated by monocytes [18]. Moreover, both monocytes and neutrophils have a recognized role in atherosclerosis, including the modulation of proatherogenic reactive oxygen species and neutrophil extracellular traps that encourage monocyte accumulation to the plaque site [19–24].

What it is about fragmentated human sleep that triggers inflammatory blood cell pathway continues to be defined. Beyond the inhibition of hypocretin production, sleep fragmentation results in hypercortisolemia [25,26]. The state of raised cortisol can prevent the inhibition of granulocyte macrophage colony-stimulating factor (GCSF) that otherwise limits neutrophil levels [27], and may therefore further increase neutrophil production [28,29].

In the broader context of public health, these data suggest that improved sleep continuity (i.e., lowering of sleep fragmentation) may offer a novel preventive strategy for lowering inflammatory status and thus lowering relative atherosclerosis risk. More broadly, these findings could help inform public health guidelines that focus on societal sleep health, one benefit of which may be lowering atherosclerotic burden.

### Limitations

A first limitation is that our analyses were constrained by the use of cross-sectional data, which precludes definitive assessment of directionality of associations. For example, it could be that cardiovascular disease (or associated treatments) may also drive sleep fragmentation in addition to, or rather than, the other way around. Although post hoc sensitivity analyses (S1 Results) indicated that incorporation of measures of cardiovascular disease did not substantively alter the significance of mediation effects in our cohort, this possibility remains. Prospective longitudinal controlled studies will be needed to directly address the issue of reverse causality.

Second, it is important to note that while the indirect mediation pathways were statistically significant, the effect sizes of the pairwise associations were overall small. This suggests that raised inflammation (our a priori study focus) is likely one of a number of possible mechanisms through which insufficient sleep contributes to atherosclerosis. Other pathways include altered autonomic nervous system activity, increased oxidative stress, impaired glucose metabolism, and endothelial dysfunction [26,30–32]. While we were unable to explore each of these mechanisms, post hoc analyses revealed that the mediation pathway was also significant when using heart rate variability (HRV) during sleep as the exposure variable in the mediation pathway—a well-established marker of the autonomic nervous system—instead of sleep fragmentation (see S1 Results). Sleep disruption is also associated with raised levels of apolipoprotein B (ApoB)—a strong predictor of cardiovascular disease [33] (though see [34]). Still, our findings indicate that one atherosclerotic mechanism in humans may involve the influence of fragmented sleep on raised inflammatory-associated neutrophil and monocyte count.

The observed associations were not significant when adjusting for the AHI measured by PSG. Sleep apnea is well known to cause sleep fragmentation, and these results are consistent with sleep apnea as a factor contributing to sleep fragmentation. While our post hoc analyses suggest that AHI per se (independently of sleep fragmentation) is not statistically significantly associated with the inflammatory-related increases in atherosclerosis, it is likely that apnea-induced cortical and autonomic arousals play a mechanistic role in this indirect association between sleep, leukocytes, and atherosclerosis.

Our reported mediation effect was stronger for neutrophils, relative to monocytes, supporting a recent study demonstrating significant indirect associations between overnight heart rate, neutrophil count, and obstructive sleep apnea [32]. Nevertheless, the current study was not powered or designed to differentiate these individual cell contributions. We propose 3 speculative, non–mutually exclusive explanations for this stronger neutrophil relationship that may warrant future investigation. First, sleep disruption is linked to a larger relative increase in neutrophils compared with monocytes [35]. Neutrophils may therefore be the more sleep sensitive—and thus important—disease-related immune cell factor of this particular pathway. Second, neutrophils are more numerous than monocytes, making up 60% to 70% of the total white blood cell count. As such, a perturbation of white blood cells (e.g., by sleep disruption) may lead to their influence being more pronounced. Third, the measure of neutrophil count is encoded as a continuous variable, whereas monocyte count is encoded as a quasi-categorical variable (see S2 Fig), which may reduce monocyte sensitivity.

While the current study accounted for common comorbidities and cofactors (e.g., insomnia and sleep apnea diagnoses, obesity, sex, age, ethnicity, smoking status, blood pressure, hypertensive medication), it must be recognized that this does not exclude the contribution of all possible comorbidities. We also cannot rule out the chance that our findings may be influenced by selection bias. The original cohort consisted of individuals free of known cardiovascular disease. This may have led to an underrepresentation of individuals with early-onset cardiovascular disease. Moreover, a small proportion (approximately 2%) of individuals were excluded from the Multi-Ethnic Study of Atherosclerosis (MESA) sleep exam due to regular continuous positive airway pressure (CPAP), oral appliance, or oxygen use, thus potentially reducing representation of those with clinically significant sleep apnea. However, the participation rate in the MESA sleep study subset was high (approximately 44%), and health profiles were generally similar between the participants who did enroll in the sleep study versus those who did not [7].

Taken together, our findings are consistent with the emerging idea of a pivotal role of neutrophils in atherogenesis [36] and establish that this association is in part mediated by sleep quality.

## Methods

### Ethics statement

Institutional review board approval was obtained at each study site, and written informed consent was obtained from all participants. This study met the Declaration of Helsinki guidelines.

### Procedure

The data were derived from the MESA Exam 5, [37], using information from its Exam 5 clinic exam and the MESA Sleep Ancillary Study, which included 1 night of home PSG, 7 consecutive days of wrist actigraphy (Actiwatch Spectrum, Philips Respironics, Murrysville, PA), and a sleep questionnaire. All participants in the main MESA were invited to participate in the additional Sleep study at Exam 5, with the exception of those regularly using CPAP or an oral device for sleep apnea. The demographic characteristics of this subset of individuals in the sleep study relative to those of the overall full study cohort have been described elsewhere (see S1 Table in [7]). Briefly, the subset of participants who enrolled in the sleep study were more likely to be younger, of nonwhite ethnicity, a nonsmoker, and normotensive compared with the MESA participants who did not enroll. Self-report doctor-diagnosed sleep apnea and other health characteristics (e.g., diabetes, obesity, myocardial infarction, asthma) were equivalent in both groups. White blood cell counts were assayed from blood from a morning blood draw the Exam 5 visit. CAC imaging from Exam 5 provided an in vivo assessment of atherosclerosis, resulting in a standard Agatston score [38]. In-depth details of the study design, sleep evaluations, blood evaluations, and CAC imaging can be found elsewhere [7,37–39].

Three validated markers of fragmented sleep were used as a priori predictor variables: (1) fragmentation index, which reflects the proportion of total sleep epochs characterized by movement, calculated separately for each night and then averaged across the 7 nights of actigraphy (see S1 Methods); (2) the number of arousals per hour of NREM sleep (the arousal index, a measure that correlates with autonomic markers of arousal [40]), estimated during the PSG night [41]; and (3) the participant self-reported sleep fragmentation (“Overall, was your typical night’s sleep during the past 4 weeks”: 0 = very sound to 4 = very restless). Second, we conducted exploratory analyses with other sleep parameters, such as actigraphy- and PSG-defined measures of sleep quality and quantity (e.g., overall duration, WASO, and, for the PSG night, percent time in each sleep stage and arousal index in REM sleep).

After removing participants with absent values on either the main predictor variables (i.e., objective and subjective measures of sleep fragmentation) and/or the main outcome variable (CAC score, or atherosclerosis Agatston score), the final sample size was 1,630 participants (752 males, mean ± SD age = 68.5 ± 9.2 years, BMI = 28.9 ± 5.5 kg/m^2^) of diverse ethnicities (602 white, 451 black, 393 Hispanic, and 184 Asian). This sample represents 34.6% of all the participants included in MESA 5 core exam (*n* = 4,716) and 72.1% of all participants that also took part in the MESA 5 sleep exam (*n* = 2,261, of which 2,060 participants had successful PSG data, 2,156 had actigraphy data, and 2,240 completed sleep questionnaires). For mediation analyses, the sample size was further reduced by removing participants with absent values on the mediator variable (e.g., the neutrophil count, final sample size, *n* = 1,110).

The hypothesis was tested using a formal mediation analysis with sleep fragmentation as the independent variable, monocyte and neutrophil counts as the mediator variables, and CAC score as the dependent variable. Specifically, the goal was to statistically determine whether monocyte and neutrophil counts could be deemed mediators of the effect of sleep fragmentation on CAC score. The relevant outcome of a formal mediation analysis is the indirect effect, which quantifies the difference between the effect of the independent variable on the dependent variable when the mediator is accounted for versus when it is not. Since both the mediators and dependent variables were continuous (S2 Fig), ordinary least squares regression was used to model direct and indirect associations. Mediation analysis was performed using the *mediation_analysis* function of the open source Pingouin statistical package for Python [42], modeled on the mediation R package [43]. As recommended for mediation analysis reporting [44], all effects were considered significant only if the 95% bias-corrected bootstrap confidence interval (of the indirect effect) was entirely above or below zero. Confidence intervals were derived from 10,000 bootstrap samples. Consistent with current guidelines, we do not report the ratio of the indirect effect over the total effect as a measure of effect size, as this ratio can be any real number and is not bounded by 0 and 1 [44].

## Supporting information

S1 Methods Supplementary methods. (DOCX)

S1 Results Supplementary results. (DOCX)

S1 Table Participants sleep parameters by atherosclerosis severity (CAC category). Data are shown as mean ± SD. *p*-Values were calculated using one-way ANOVA for continuous variables and chi-squared test of independence for categorical variables. Statistical significance, *p* < 0.05. AHI, apnea-hypopnea index; AI, arousal index; CAC, coronary artery calcification; WASO, wake after sleep onset.
(DOCX)

S1 Fig Bivariate regression plots. (TIF)

S2 Fig Distribution (kernel density estimation) plots of the main variables. (TIF)

## Abbreviations

AHI: apnea-hypopnea index
ApoB: apolipoprotein B
BMI: body mass index
CAC: Coronary Artery Calcification
CPAP: continuous positive airway pressure
DBP: diastolic blood pressure
HRV: heart rate variability
MESA: Multi-Ethnic Study of Atherosclerosis
NREM: non-rapid eye movement
PSG: polysomnography
SBP: systolic blood pressure
WASO: wake after sleep onset
