# Supplementary material for: Broken sleep predicts hardened blood vessels
Source: PLoS Biol. 2020 Jun 4;18(6):e3000726. doi: 10.1371/journal.pbio.3000726 (PMC7271997; doi:10.1371/journal.pbio.3000726)
Supplement: S1 Methods — (DOCX) [file pbio.3000726.s001.docx]

# Supplementary Methods

Described in full in the official documentation of the MESA website^[[1]](#footnote-1)^, as well as the National Sleep Research Resource^[[2]](#footnote-2)^, and adapted here from the text of those sources, the Multi-Ethnic Study of Atherosclerosis (MESA) is a multi-center prospective study of more than 6,000 ethnically diverse men and women aged 45-84 from six communities in the United States. MESA was designed to investigate the prevalence and progression of subclinical cardiovascular disease (CVD) as well as to identify CVD risk factors predicting the development of clinically overt CVD in an ethnically diverse population [(Bild et al., 2002)](https://paperpile.com/c/7e0Dtd/qNes).

There have been four follow-up exams to date since the initial exam, in the years 2003-2004 (Exam 2), 2004-2005 (Exam 3), 2005-2007 (Exam 4), and 2010-2012 (Exam 5). The MESA 5 Core exam occurred from April 2010 to February 2012, 10 years after the initial exam. Similar to the prior follow-up exams, Exam 5 collected interval medical history, anthropometrics, blood pressure readings, fasting venipuncture, spot urine collection, nutrition and physical activity surveys, smoking history, ankle/arm index, retinal photography, and ECG. In addition, cardiac MRI was repeated in participants who underwent cardiac MRI at exam 1, and cognitive function testing was newly performed in all MESA Exam 5 participants. Randomly selected participants were invited to participate in the MESA ancillary study (70% of the MESA cohort), which performed cardiac CT imaging for measurement of CAC.

All MESA participants were also invited to participate in the MESA Sleep ancillary study at MESA Exam 5 (2010-2013). Sleep exams were scheduled to occur after the MESA 5 core exam. The purpose of MESA Sleep was to obtain quantitative measures of sleep and sleep-disordered breathing (SDB) to better characterize specific sleep traits and sleep disorders and their CVD risk associations across ethnic groups, as well as to determine the association of sleep indices with incident CVD. The sleep protocol included one night of home polysomnography (PSG), seven consecutive days of wrist actigraphy (Actiwatch Spectrum, Philips Respironics, Murrysville, PA), and a sleep questionnaire.

In-home polysomnography (PSG) was performed using the Compumedics Somte System (Compumedics LTd., Abbostville, Australia. The recording montage consisted of cortical EEG (C4-M1, Oz-Cz, and Fz-Cz channels), bilateral EOG, chin EMG, thoracic and abdominal respiratory inductance plethysmography (by auto-calibrating inductance bands); airflow (by nasal-oral thermocouple and pressure recording from a nasal cannula); ECG; leg movements, and finger pulse oximetry. EEG, EOG, EMG and ECG were all sampled at 256 Hz. Nocturnal recordings were transmitted to the centralized reading center at Brigham and Women’s Hospital and data were scored by trained technicians using current guidelines [(Iber, 2007; Silber et al., 2007)](https://paperpile.com/c/7e0Dtd/6fsV+hUFr).

Actigraphy was performed using the Actiwatch Spectrum wrist actigraph (Philips Respironics, Murrysville, PA) worn on the participant’s non-dominant wrist. Output was sent to the Sleep Reading Center at Brigham and Women’s Hospital where records were scored with use of the corresponding sleep diary. Specifically, actigraphy data were aggregated in 30-second epochs and automatically scored as sleep or wake by a validated algorithm implemented in the Actiware-Sleep v.5.59 analysis software (Mini Mitter Co., Inc.), after manually editing the sleep period using sleep diary data and event and light markers. Two scorers scored MESA actigraphy studies. Intra-scorer reliability for average sleep duration, sleep efficiency, and WASO were 0.91, 0.97, and 0.91, respectively. Sleep fragmentation was defined as the sum percent mobile epochs and percent immobile bouts less than 1-minute duration to the number of immobile bouts, for the given interval. This is also known as the restlessness index or movement and fragmentation index.

White blood cells (WBC) were assessed in blood samples collected at Exam 5 at a central laboratory. Blood assays included total WBC count, and leukocyte subsets (basophils, eosinophils, neutrophils, lymphocytes, and monocytes) were determined as complete blood count with differential analysis.

Mediation models were adjusted for factors known to affect cardiovascular risk, specifically age, sex, race/ethnicity, body mass index (BMI; in kg/m^2^), smoking status, use of antihypertensive medication, blood pressure as well as medical diagnosis of sleep apnea and insomnia. Information on doctor-diagnosed sleep apnea/insomnia was obtained from the sleep questionnaire survey: *“Have you been told by a doctor that you have any of the following:? a) Sleep Apnea (or obstructive sleep apnea, OSA) b) Insomnia”*. Those who answered ‘yes’ were defined as having doctor-diagnosed sleep apnea and / or insomnia, respectively. Smoking was defined as never, former (no smoking within the past 30 days), or current. Resting blood pressure was measured three times in the seated position and the average of the second and the third served as systolic and diastolic blood pressure.

# References

[Bild, D. E., Bluemke, D. A., Burke, G. L., Detrano, R., Diez Roux, A. V., Folsom, A. R., Greenland, P., Jacob, D. R., Jr, Kronmal, R., Liu, K., Nelson, J. C., O’Leary, D., Saad, M. F., Shea, S., Szklo, M., & Tracy, R. P. (2002). Multi-Ethnic Study of Atherosclerosis: objectives and design. *American Journal of Epidemiology*, *156*(9), 871–881.](http://paperpile.com/b/7e0Dtd/qNes) <https://www.ncbi.nlm.nih.gov/pubmed/12397006>

[Iber, C. (2007). *The AASM manual for the scoring of sleep and associated events: rules, terminology and technical specifications*. American Academy of Sleep Medicine.](http://paperpile.com/b/7e0Dtd/6fsV)

[Silber, M. H., Ancoli-Israel, S., Bonnet, M. H., Chokroverty, S., Grigg-Damberger, M. M., Hirshkowitz, M., Kapen, S., Keenan, S. A., Kryger, M. H., Penzel, T., Pressman, M. R., & Iber, C. (2007). The visual scoring of sleep in adults. *Journal of Clinical Sleep Medicine: JCSM: Official Publication of the American Academy of Sleep Medicine*, *3*(2), 121–131.](http://paperpile.com/b/7e0Dtd/hUFr) <https://www.ncbi.nlm.nih.gov/pubmed/17557422>

1. https://www.mesa-nhlbi.org/ [↑](#footnote-ref-1)
2. https://sleepdata.org/datasets/mesa [↑](#footnote-ref-2)
