# Supplementary material for: Broken sleep predicts hardened blood vessels
Source: PLoS Biol. 2020 Jun 4;18(6):e3000726. doi: 10.1371/journal.pbio.3000726 (PMC7271997; doi:10.1371/journal.pbio.3000726)
Supplement: S1 Results — (DOCX) [file pbio.3000726.s002.docx]

# Supplementary Results

Importantly, our analyses were limited by the use of cross-sectional data, and while a causal mediation framework was used, this precludes definitive assessment of directionality of associations. The rationale for interpreting our findings in the specific direction of sleep leading to atherosclerosis is twofold. First, the present article was principally motivated by directionally specific findings in rodents. In those models, the authors used the causal manipulation of sleep fragmented sleep in otherwise healthy animals without pre-existing atherosclerosis, which resulted in raised inflammatory blood cell markers that, in turn, led to the development of atherosclerotic plaques [(McAlpine et al., 2019)](https://paperpile.com/c/7e0Dtd/Mne2). Second, our analyses focused on CAC, and not cardiovascular disease (CVD), as the outcome variable. While CAC is one of the most well-established predictors of future cardiovascular disease, a high CAC score per se is not deterministic of present-state CVD and/or associated treatments. In the current study, we therefore wanted to test the experimental hypothesis by looking at CAC relationships in the early inception stages of CVD (i.e., subclinical atherosclerosis) in an effort to minimize the issue of reverse causality to a degree.

To empirically address this point in a more direct manner, we conducted mediation analyses that specifically excluded participants with a history of cardiovascular events, whilst adjusting for the same covariates that were included in our full cohort analysis. The main mediation between actigraphy-defined sleep fragmentation, neutrophil counts and atherosclerosis remained significant when excluding participants with history of congestive heart failure (𝛽=0.44, 95% CIs=0.03-1.34), peripheral vascular disease (𝛽=0.41, 95% CIs=0.002-1.26), stroke (𝛽=0.39, 95% CIs=0.03-1.23; and with trend significance when removing participants with history of myocardial infarction: 𝛽=0.36, 95% CIs=-0.004-1.08). Furthermore, two-sided Welch’s t-test comparing the actigraphy and PSG sleep fragmentation of participants with or without history of cardiovascular events yielded no significant differences for any of the outcomes considered (all p’s > 0.3). Altogether, this set of post-hoc analyses tentatively suggests that a history of cardiovascular events is a less parsimonious factor driving sleep fragmentation in this specific cohort. Parenthetically, data has also indicated that reductions in sleep fragmentation (by means of CPAP treatment in apnea patients) are associated with decreased atherosclerosis (Drager et al. 2007), suggesting at least a partial mechanistic and directionally specific role of sleep fragmentation in atherosclerosis risk.

In a similar fashion, we conducted mediation analyses that specifically excluded participants with an absolute neutrophils count below 1.5x109/L: a standard threshold to define mild neutropenia (Haddy et al., 1999). The main mediation effect remained significant, which would suggest that conditions associated with neutropenia, such as benign ethnic neutropenia, are less likely to be a significant confound in our data.

We also conducted post-hoc analyses to determine whether additional markers of autonomic arousal during sleep could be associated with increased subclinical atherosclerosis via higher counts of neutrophil/monocyte. We specifically focused on well-established measures of heart rate variability (HRV). After adjusting for the same covariates that were included in our main mediation analysis, we indeed found that worse HRV outcomes (reflecting lower parasympathetic tone), both in the time and frequency domains, were significantly associated with an increase in neutrophil counts, which in turn predicted a higher CAC score.

First, we found that a lower log-transformed absolute spectral power of all normal-to-normal (NN) intervals between 0.003 and 0.04 Hz (i.e. very low frequency power, or VLF), calculated across the entire night of polysomnography-recorded sleep, predicted a higher CAC score, via an increase in neutrophil counts (n=932, 𝛽=-4.56, 95% CIs=-12.6--0.70). Similarly, a lower percentage of differences between adjacent NN intervals greater than 10 and 20 ms (pNN10 and pNN20, respectively), calculated across the entire night of polysomnography-recorded sleep, was significantly associated with higher CAC, via a raise in neutrophil counts (n=932, pNN10: 𝛽=-0.24, 95% CIs=-0.59--0.05; pNN20: 𝛽=-0.16, 95% CIs=-0.41--0.03).

Decreased VLF power has been associated with higher levels of inflammation and higher cardiac mortality [(Shaffer & Ginsberg, 2017)](https://paperpile.com/c/7e0Dtd/89d3). The pNNx family is a well-known marker of parasympathetic activity, with higher values being associated with lower cardiovascular disease risk [(Mietus et al., 2002)](https://paperpile.com/c/7e0Dtd/z8At). These additional post-hoc analyses therefore help support the proposal that one potential pathway through which sleep fragmentation may raise inflammatory-related white blood cells and thus atherosclerosis risk is autonomic dysfunction. Such data lead to the testable hypothesis that the measurement of HRV during sleep e.g., by using wearable photoplethysmography sensors, is sensitive enough to detect this inflammatory-related increased risk for atherosclerosis.

Finally, to determine whether our main mediation pathways between sleep fragmentation, neutrophils/monocytes, and atherosclerosis were sex-specific, we conducted post-hoc analysis specifically for males and females. While not adequately powered, we did not find any significant mediation effect between sleep fragmentation, neutrophils/monocytes, and atherosclerosis for either sex when adjusting for all the covariates (excluding sex). Interestingly, unadjusted models did reveal a significant mediation in females only between the average actigraphy fragmentation, neutrophil count, and CAC score (n=595, 𝛽=0.81, 95% CIs=0.13-2.15), suggesting that the neutrophil-related association between sleep fragmentation and CAC might be somewhat stronger in females than in males.

# References

Drager, L. F., Bortolotto, L. A., Figueiredo, A. C., Krieger, E. M., & Lorenzi, G. F. (2007). Effects of continuous positive airway pressure on early signs of atherosclerosis in obstructive sleep apnea. American Journal of Respiratory and Critical Care Medicine, 176(7), 706–712. https://doi.org/10.1164/rccm.200703-500OC

Haddy, T. B., Rana, S. R., & Castro, O. (1999). Benign ethnic neutropenia: what is a normal absolute neutrophil count? The Journal of Laboratory and Clinical Medicine, 133(1), 15–22. https://doi.org/10.1053/lc.1999.v133.a94931

[McAlpine, C. S., Kiss, M. G., Rattik, S., He, S., Vassalli, A., Valet, C., Anzai, A., Chan, C. T., Mindur, J. E., Kahles, F., Poller, W. C., Frodermann, V., Fenn, A. M., Gregory, A. F., Halle, L., Iwamoto, Y., Hoyer, F. F., Binder, C. J., Libby, P., … Swirski, F. K. (2019). Sleep modulates haematopoiesis and protects against atherosclerosis. *Nature*. https://doi.org/](http://paperpile.com/b/7e0Dtd/Mne2)[10.1038/s41586-019-0948-2](http://dx.doi.org/10.1038/s41586-019-0948-2)

[Mietus, J. E., Peng, C.-K., Henry, I., Goldsmith, R. L., & Goldberger, A. L. (2002). The pNNx files: re-examining a widely used heart rate variability measure. *Heart* , *88*(4), 378–380. https://doi.org/](http://paperpile.com/b/7e0Dtd/z8At)[10.1136/heart.88.4.378](http://dx.doi.org/10.1136/heart.88.4.378)

[Shaffer, F., & Ginsberg, J. P. (2017). An Overview of Heart Rate Variability Metrics and Norms. *Frontiers in Public Health*, *5*, 258. https://doi.org/](http://paperpile.com/b/7e0Dtd/89d3)[10.3389/fpubh.2017.00258](http://dx.doi.org/10.3389/fpubh.2017.00258)
