# Supplementary material for: Broken sleep predicts hardened blood vessels
Source: PLoS Biol. 2020 Jun 4;18(6):e3000726. doi: 10.1371/journal.pbio.3000726 (PMC7271997; doi:10.1371/journal.pbio.3000726)
Supplement: S1 Table — Data are shown as mean ± SD. p-Values were calculated using one-way ANOVA for continuous variables and chi-squared test of independence for categorical variables. Statistical significance, p < 0.05. AHI, apnea-hypopnea index; AI, arousal index; CAC, coronary artery calcification; WASO, wake after sleep onset. (DOCX) [file pbio.3000726.s003.docx]

|  | Very low (CAC = 0) | Low (CAC = 1 - 100) | High (CAC = 101 - 400) | Very high (CAC > 401) | *p* |
| --- | --- | --- | --- | --- | --- |
| ***Actigraphy*** | | | | | |
| Fragmentation (%) | 18.5 ± 6.2 | 19.9 ± 6.4 | 21.0 ± 6.8 | 21.7 ± 7.4 | ***<.001*** |
| Efficiency (%) | 90.1 ± 3.4 | 89.8 ± 3.3 | 89.4 ± 4.0 | 89.7 ± 3.8 | ***0.047*** |
| Total Sleep Time (min) | 393.2 ± 72.1 | 387.9 ± 78.3 | 378.0 ± 91.2 | 402.5 ± 86.2 | ***0.002*** |
| WASO (min) | 36.4 ± 15.8 | 37.2 ± 15.6 | 37.9 ± 17.6 | 39.3 ± 17.6 | *0.106* |
| ***Polysomnography*** | | | | | |
| Total Sleep Time (min) | 371.5 ± 80.2 | 367.3 ± 81.0 | 340.4 ± 80.2 | 360.8 ± 77.7 | ***<.001*** |
| Sleep Efficiency (%) | 78.0 ± 13.2 | 76.8 ± 12.6 | 72.5 ± 14.5 | 74.7 ± 13.2 | ***<.001*** |
| N1 sleep (%) | 12.5 ± 8.4 | 14.1 ± 8.1 | 15.1 ± 9.6 | 17.5 ± 10.4 | ***<.001*** |
| N2 sleep (%) | 57.3 ± 10.0 | 56.9 ± 9.8 | 57.6 ± 10.9 | 58.4 ± 10.5 | *0.289* |
| N3 sleep (%) | 11.3 ± 9.2 | 10.0 ± 8.8 | 10.2 ± 9.7 | 7.6 ± 7.7 | ***<.001*** |
| REM sleep (%) | 18.9 ± 6.6 | 19.0 ± 6.6 | 17.0 ± 6.9 | 16.5 ± 6.5 | ***<.001*** |
| WASO (min) | 82.5 ± 62.5 | 92.1 ± 60.6 | 109.1 ± 74.9 | 104.0 ± 66.1 | ***<.001*** |
| AI (all) | 20.2 ± 11.2 | 21.9 ± 11.6 | 23.3 ± 12.6 | 24.5 ± 12.5 | ***<.001*** |
| AI (REM) | 17.0 ± 11.2 | 18.0 ± 11.8 | 17.9 ± 12.7 | 17.4 ± 11.7 | *0.580* |
| AI (NREM) | 20.8 ± 12.0 | 22.7 ± 12.3 | 24.3 ± 13.4 | 25.8 ± 13.5 | ***<.001*** |
| AHI | 20.0 ± 17.4 | 24.5 ± 20.3 | 25.3 ± 19.0 | 26.8 ± 19.4 | ***<.001*** |
| REM latency | 105.7 ± 72.9 | 103.8 ± 74.7 | 119.1 ± 82.8 | 110.4 ± 76.2 | ***0.039*** |

**S1 Table. Participants sleep parameters by atherosclerosis severity (CAC category)**. Data are shown as mean ± SD. P-values were calculated using one-way ANOVA for continuous variables and chi-squared test of independence for categorical variables. Significant p-values are denoted in bold. WASO, wake after sleep onset; AI, arousal index; AHI, apnea-hypopnea index. Statistical significance, p < 0.05.
