# Supplementary figures and images for: Broken sleep predicts hardened blood vessels
Source: PLoS Biol. 2020 Jun 4;18(6):e3000726. doi: 10.1371/journal.pbio.3000726 (PMC7271997; doi:10.1371/journal.pbio.3000726)

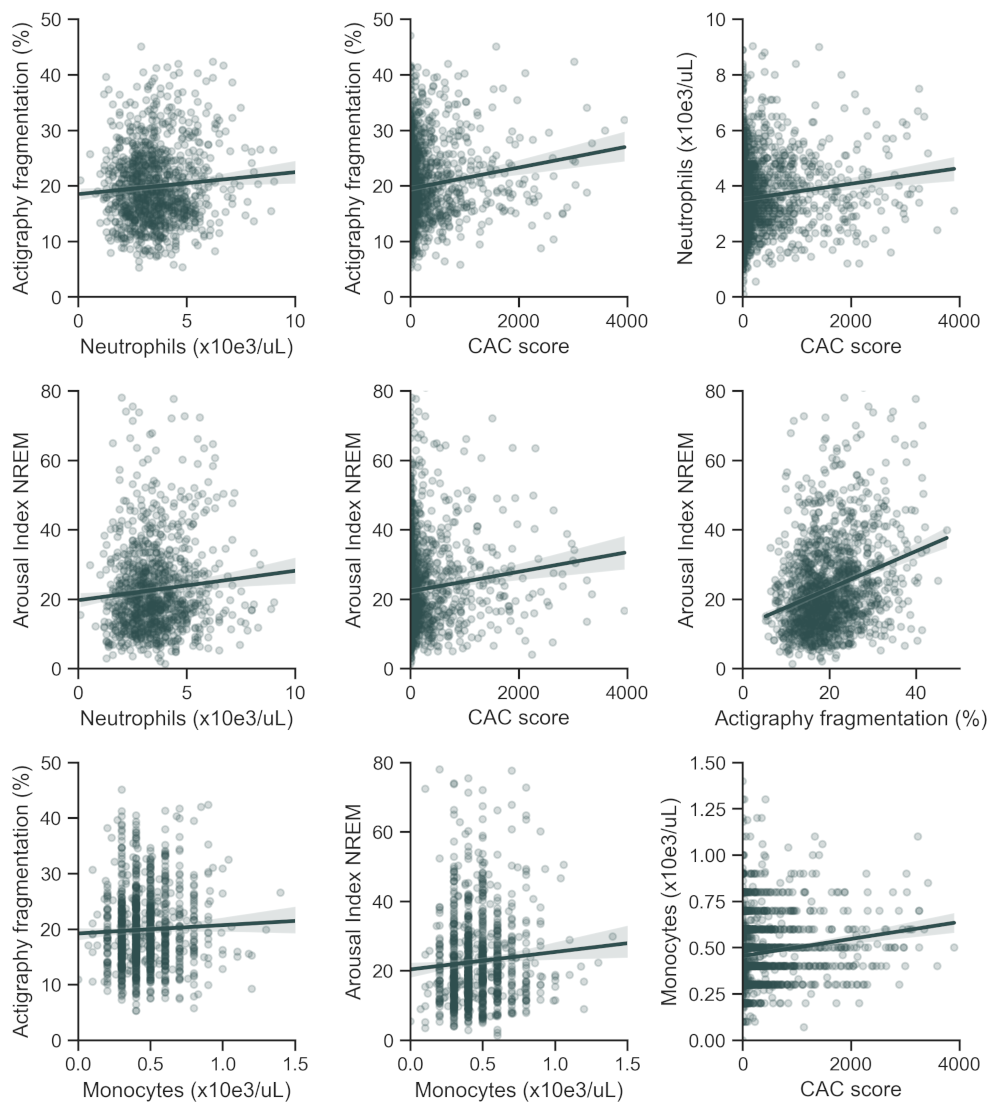

Supplement: S1 Fig — (TIF) [file pbio.3000726.s004.tif]

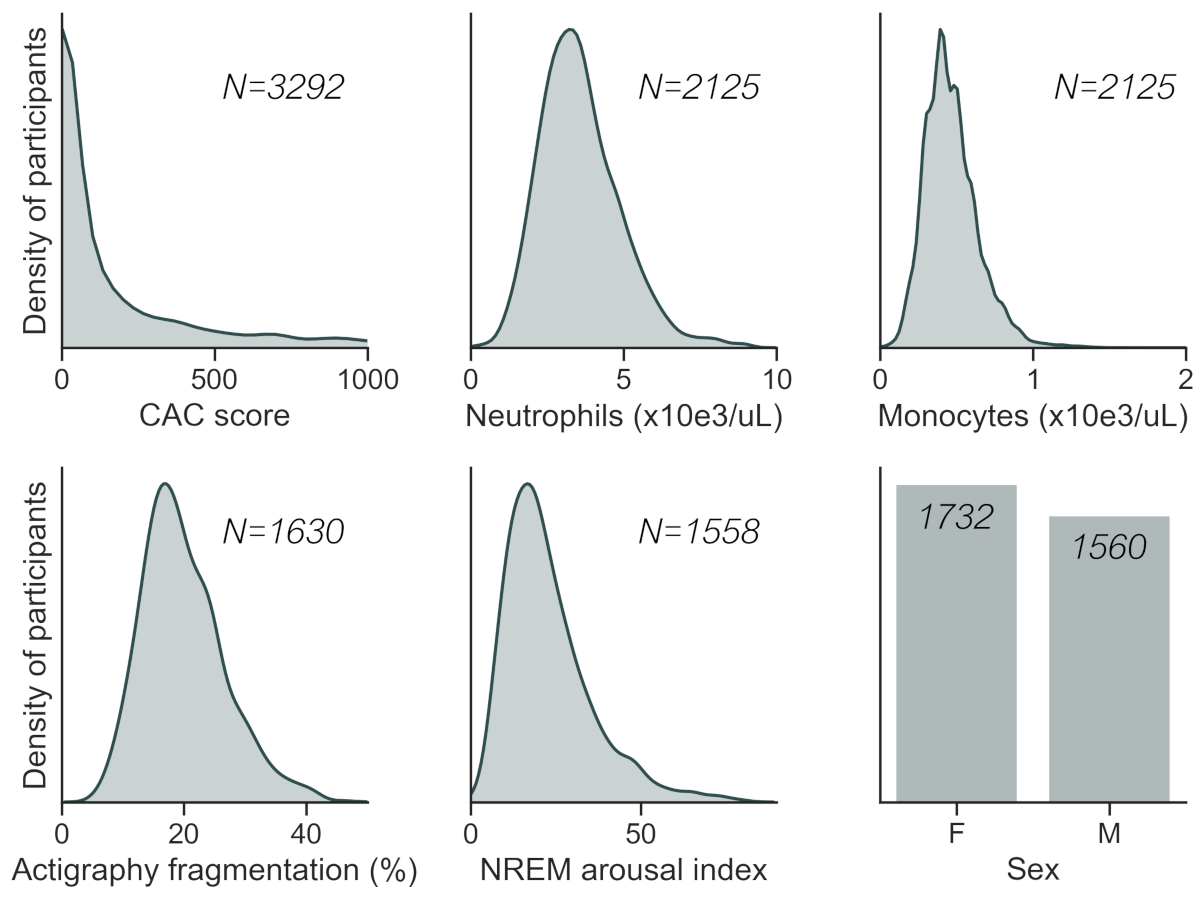

Supplement: S2 Fig — (TIF) [file pbio.3000726.s005.tif]
